# Supplementary material for: Effects of scent lure on camera trap detections vary across mammalian predator and prey species
Source: PLoS One. 2020 May 12;15(5):e0229055. doi: 10.1371/journal.pone.0229055 (PMC7217433; doi:10.1371/journal.pone.0229055)
Supplement: S1 Table — (PDF) [file pone.0229055.s001.pdf]

S1 Table. Summary of camera trap detections. Total of 14,760 detections (Dtn) across 31 mammal species or groups over the study period, shown by species and year. Carnivores were classified as large or small according to a body mass threshold of 20 kg. Detection rate is the total number of detections divided by camera effort (120 days * 844 cameras) *1000. Species highlighted in bold were assessed in species-specific models.

Group	Common Name	Latin Name	2015 Dtn	2016 Dtn	Total Dtn	Dtn Rate
Large Carnivore	Cougar	*Puma concolor*	10	1	11	0.11
	**Gray Wolf**	***Canis lupus***	**40**	**33**	**73**	**0.72**
	Black Bear	*Ursus americanus*	307	428	735	7.26
Small Carnivore	Foxes	*Vulpes spp.*	0	1	1	0.01
	Wild Boar	*Sus scrofa*	1	0	1	0.01
	Bobcat	*Lynx rufus*	1	0	1	0.01
	Weasels and Ermine	*Mustela spp.*	0	4	4	0.04
	Mink	*Neovision vision*	4	0	4	0.04
	Raccoon	*Procyon lotor*	8	9	17	0.17
	Striped Skunk	*Mephitis mephitis*	37	50	87	0.86
	Wolverine	*Gulo gulo*	22	2	24	0.24
	Marten	*Martes americana*	46	22	68	0.67
	Badger	*Taxidea taxus*	28	46	74	0.73
	**Fisher**	***Pekania pennanti***	**54**	**51**	**105**	**1.04**
	Red fox	*Vulpes vulpes*	113	61	174	1.72
	Canada Lynx	*Lynx canadiensis*	121	80	201	1.98
	Coyote	*Canis latrans*	1082	925	2007	19.82
Small Mammal	Ground squirrel	*Urocitellus sp.*	0	1	1	0.01
	Voles, Mice and Allies	*Muridae spp.*	2	1	3	0.03
	Groundhog	*Marmota monax*	3	1	4	0.04
	**Richardson's Gr. Squirrel**	***Urocitellus richardsonii***	**32**	**102**	**134**	**1.32**
	Red Squirrel	*Tamiasciurus hudsonicus*	36	239	275	2.72
	White-tailed Jack Rabbit	*Lepus townsendii*	156	247	403	3.98
	Snowshoe Hare	*Lepus americanus*	332	397	729	7.20
Ungulate	Bison	*Bison bison*	16	0	16	0.16
	Woodland Caribou	*Rangifer tarandus*	30	37	67	0.66
	Elk (wapiti)	*Cervus canadensis*	351	73	424	4.19
	Deer	*Odocoileus spp.*	197	343	540	5.33
	**Moose**	***Alces alces***	**365**	**281**	**646**	**6.38**
	Mule deer	*Odocoileus hemionus*	1368	1285	2653	26.19
	White-tailed Deer	*Odocoileus virgianus*	3284	1994	5278	52.11
