# Supplementary material for: Effects of scent lure on camera trap detections vary across mammalian predator and prey species
Source: PLoS One. 2020 May 12;15(5):e0229055. doi: 10.1371/journal.pone.0229055 (PMC7217433; doi:10.1371/journal.pone.0229055)
Supplement: S2 Table — (PDF) [file pone.0229055.s002.pdf]

S2 Table. Habitat types and their frequencies across camera trap stations. Habitat types were categorized following Alberta Biodiversity Monitoring Institute field protocols described in [22]. We grouped habitat types into forest (n = 315 camera stations), grassland (n = 268), and wetland (n = 261), with scent lure deployed in roughly half of each broad habitat category. Stations scored as “bare” and “water” were excluded from our analyses due to small sample sizes.

	Forest		Grassland		Wetland	
**Habitat Type**	**No Lure**	**Lure**	**No Lure**	**Lure**	**No Lure**	**Lure**
Alkali					1	
Bare						
Deciduous	82	74				
GraminoidFen					3	3
GrassHerb			134	134		
Marsh					14	15
Mixedwood	2	24				
Pine	27	24				
Shrub	14	9				
ShrubbyBog					5	2
ShrubbyFen					7	6
ShrubbySwamp					11	15
Spruce	24	17				
TreedBog-BlackSpruce					53	41
TreedFen-BlackSpruce						
TreedFen-Larch					3	27
TreedFen-Mixedwood					6	5
TreedSwamp-Deciduous					7	8
TreedSwamp-Spruce					2	
TreedWetland-Mixedwood						
Water						
Total Camera Stations	167	148	134	134	139	122
