# Supplementary material for: Effects of scent lure on camera trap detections vary across mammalian predator and prey species
Source: PLoS One. 2020 May 12;15(5):e0229055. doi: 10.1371/journal.pone.0229055 (PMC7217433; doi:10.1371/journal.pone.0229055)
Supplement: S3 Table — (PDF) [file pone.0229055.s003.pdf]

**S3 Table. Human disturbance types and their frequencies across camera trap stations.** Feature types associated with human disturbance were categorized following the Alberta Biodiversity Monitoring Institute protocols [22]. We classified camera trap stations as “disturbed” if they were scored as one of these features types. Approximately half of the 249 camera stations classified as “disturbed” were deployed with scent lure.

| Disturbance Feature Type | No Lure | Lure |
|---|---|---|
| ACREAGE | 3 | |
| AGRICULTURE_CLEARING | 3 | 7 |
| CROP | | 1 |
| CULTIVATION | 80 | 72 |
| CUTBLOCK | 8 | 9 |
| CUTLINE-TRAIL | 4 | 3 |
| DUGOUT | | 1 |
| IND-LOW | 1 | 4 |
| PIPELINE | | |
| ROAD-GRAVEL-1L | 2 | |
| ROAD-PAVED-UNDIV-2L | 1 | |
| ROAD-UNIMPROVED | 2 | 1 |
| RURAL_1-5ha | 7 | |
| RURAL_GT_5ha | 1 | |
| RURAL_LESS_1ha | 1 | |
| RURAL-RESIDENCE | 1 | |
| SOFT_ROAD-GRAVEL-1L | 9 | 6 |
| SOFT_ROAD-GRAVEL-2L | 2 | |
| SOFT_ROAD-PAVED-UNDIV-2L | 3 | 2 |
| TAME_PASTURE | 3 | 3 |
| TRANS-LINE | 2 | 1 |
| TRUCK-TRAIL | 1 | 1 |
| URBAN_RESIDENCE | 1 | |
| VEGETATED-EDGE-ROADS | | |
| WELL | | 2 |
| WELL-BIT | | |
| WELL-GAS | | 1 |
| WELL-OIL | | |
| Total Camera Stations | 135 | 114 |
