# Supplementary material for: Effects of scent lure on camera trap detections vary across mammalian predator and prey species
Source: PLoS One. 2020 May 12;15(5):e0229055. doi: 10.1371/journal.pone.0229055 (PMC7217433; doi:10.1371/journal.pone.0229055)
Supplement: S4 Table — (PDF) [file pone.0229055.s004.pdf]

S4 Table. AIC model selection for candidate generalized linear mixed models. Candidate zero-inflated negative binomial generalized linear mixed models considered in evaluating the effects of scent lure on camera trap detections of mammal species in Alberta, Canada. The total number of detections across all species was the response variable. The number of model parameters is given by *k*; all models included a random effect for site (to account for non-independence among clusters of 4 cameras per site). Models were ranked by Akaike Information Criterion (AIC). The top model (lowest AIC) was significantly better than the second ranked model based on a Chi square test ($\chi^2 = 34.8$, df = 1, $p < 0.001$) and was subsequently used to model detections of sub-groups of species.

**Model predictor variables**	***k***	**AIC**	**ΔAIC**
Lure + Habitat	7	6172.3	0
Lure + Habitat + Disturbance	8	6174.3	2.0
Lure	5	6203.3	31.0
Lure + Disturbance	6	6205.1	32.8
Null	3	6226.5	54.2
