# Supplementary material for: Effects of scent lure on camera trap detections vary across mammalian predator and prey species
Source: PLoS One. 2020 May 12;15(5):e0229055. doi: 10.1371/journal.pone.0229055 (PMC7217433; doi:10.1371/journal.pone.0229055)
Supplement: S5 Table — (PDF) [file pone.0229055.s005.pdf]

S5 Table. Parameter estimates for fixed effects from generalised linear mixed models. Models regressing camera trap detections against predictor variables for Lure and Habitat were run on different subsets of the data (all data, or by species group or species listed in the Model column). The reference categories were No Lure (vs. Lure) and Forest (vs. Grassland, Wetland, or Non-Forest).

**Model**	**Variable**	**Estimate**	**Std. Error**	**Z value**	***p***
All	(Intercept)	2.44	0.09	27.82	0.000
	Lure	0.29	0.06	4.82	0.000
	Grassland	0.10	0.12	0.88	0.380
	Wetland	-0.52	0.10	-5.35	0.000
Predators	(Intercept)	1.00	0.09	11.23	0.000
	Lure	0.75	0.07	10.45	0.000
	Grassland	-0.18	0.12	-1.51	0.131
	Wetland	-0.55	0.11	-5.18	0.000
Prey	(Intercept)	2.00	0.12	17.29	0.000
	Lure	0.02	0.07	0.27	0.789
	Grassland	0.17	0.15	1.10	0.273
	Wetland	-0.49	0.12	-3.97	0.000

**Model**	**Variable**	**Estimate**	**Std. Error**	**Z value**	***p***
Large Carnivores	(Intercept)	-0.66	0.18	-3.71	0.000
	Lure	0.81	0.13	6.09	0.000
	Grassland	-3.81	0.42	-9.16	0.000
	Wetland	-0.50	0.17	-2.97	0.003
Wolf	(Intercept)	-4.00	0.84	-4.76	0.000
	Lure	-0.01	0.34	-0.03	0.973
	Grassland	-2.12	0.86	-2.46	0.014
	Wetland	0.04	0.43	0.09	0.931
Small Carnivores	(Intercept)	0.32	0.11	2.88	0.004
	Lure	0.78	0.08	9.46	0.000
	Grassland	0.33	0.14	2.33	0.020
	Wetland	-0.42	0.13	-3.13	0.002
Fisher	(Intercept)	-4.85	0.68	-7.13	0.000
	Scent-Lure	2.23	0.36	6.17	0.000
	Non-Forest	-0.59	0.35	-1.67	0.095

**Model**	**Variable**	**Estimate**	**Std. Error**	**Z value**	***p***
Small Mammals	(Intercept)	-0.38	0.23	-1.68	0.093
	Scent-Lure	0.29	0.17	1.75	0.080
	Grassland	0.36	0.27	1.33	0.183
	Wetland	-0.06	0.24	-0.25	0.802
Richardson Ground Squirrel	(Intercept)	-3.60	1.19	-3.04	0.002
	Scent-Lure	0.00	1.14	0.00	0.998
	Non-Forest	1.45	1.24	1.17	0.241
Ungulates	(Intercept)	1.40	0.15	9.29	0.000
	Scent-Lure	-0.03	0.08	-0.41	0.680
	Grassland	0.13	0.18	0.74	0.458
	Wetland	-0.43	0.14	-2.95	0.003
Moose	(Intercept)	-1.23	0.22	-5.68	0.000
	Scent-Lure	0.07	0.15	0.49	0.621
	Grassland	-1.03	0.27	-3.85	0.000
	Wetland	0.00	0.21	0.00	0.999
