# Supplementary material for: Effects of scent lure on camera trap detections vary across mammalian predator and prey species
Source: PLoS One. 2020 May 12;15(5):e0229055. doi: 10.1371/journal.pone.0229055 (PMC7217433; doi:10.1371/journal.pone.0229055)
Supplement: Attachment — Submitted filename: Response to Reviewers.docx [file pone.0229055.s006.docx]

**Response to Reviewers, Holinda et al., PONE-D-20-02366**

Dear Dr. Mousseau,

We thank you and the reviewer for your thoughtful feedback.

We have addressed each comment below, highlighting our responses in *blue italic font*, using line numbers that correspond to the Revised Manuscript with Track Changes.

As directed, we have also discussed data availability in our revised cover letter.

We hope that you find our revisions satisfactory, and our revised manuscript suitable for publication in *PLOS ONE*.

Sincerely,

Cole Burton and co-authors

PONE-D-20-02366
Effects of scent lure on camera trap detections vary across mammalian predator and prey species
PLOS ONE

Dear Dr Burton,

Thank you for submitting your manuscript to PLOS ONE. After careful consideration, we feel that it has great merit and will be acceptable following a few very minor edits and a few changes mostly related to the statistical analysis. As someone who uses this approach, I found you paper very well developed and likely to be of great use for those wishing to employ camera traps in their research. Therefore, we invite you to submit a revised version of the manuscript that addresses the points raised during the review process.

Reviewer #1 raises several important issues including data availability, statistical analyses, presentation of models and results,  and comparison to occupancy models. Please address these issues in your revision. 


When submitting your revision, we need you to address these additional requirements:

1. Please ensure that your manuscript meets PLOS ONE's style requirements, including those for file naming.

*We have formatted our manuscript and file names to meet PLOS ONE's style requirements.*

2. In your Methods section, please provide additional location information of the study area, including geographic coordinates for the data set if available.

*We added geographic coordinates to the Methods section (lines 101-102: “CT sites extended from 49.05 to 60.00 decimal degrees latitude, and -110.14 to -117.67 longitude”).*

3. We note that you have indicated that data from this study are available upon request. PLOS only allows data to be available upon request if there are legal or ethical restrictions on sharing data publicly. For more information on unacceptable data access restrictions, please see <http://journals.plos.org/plosone/s/data-availability#loc-unacceptable-data-access-restrictions>.

*Data from this study were provided by a third party, the Alberta Biodiversity Monitoring Institute (ABMI), under a Data Sharing Agreement that does not permit us to redistribute the data. The camera trap detection data may be obtained directly from the ABMI website (*[*https://abmi.ca/home/data-analytics/da-top/da-product-overview/remote-camera-mammal-data/remote-camera-mammal-data-download.html*](https://abmi.ca/home/data-analytics/da-top/da-product-overview/remote-camera-mammal-data/remote-camera-mammal-data-download.html)*). The ABMI does not release the exact locations of their monitoring locations (as part of their policy designed to protect the integrity of their long-term monitoring sites), but the data on habitat (vegetation) and disturbance (human footprint) at camera stations can be obtained by request from the ABMI (*[*https://abmi.ca*](https://abmi.ca)*), specifically the Director of the Information Centre (Tara Narwani, 780-492-5531,* [*tnarwani@ualberta.ca*](mailto:tnarwani@ualberta.ca)*,* [*https://www.abmi.ca/home/contact-us/abmi-directory.html*](https://www.abmi.ca/home/contact-us/abmi-directory.html)*).*

*We have also included this information in a revised cover letter, as requested.*

4. We note that Figure 1 in your submission contain map images which may be copyrighted. All PLOS content is published under the Creative Commons Attribution License (CC BY 4.0), which means that the manuscript, images, and Supporting Information files will be freely available online, and any third party is permitted to access, download, copy, distribute, and use these materials in any way, even commercially, with proper attribution. For these reasons, we cannot publish previously copyrighted maps or satellite images created using proprietary data, such as Google software (Google Maps, Street View, and Earth). For more information, see our copyright guidelines: <http://journals.plos.org/plosone/s/licenses-and-copyright>.

*There are no copyrighted map images in Figure 1 of our manuscript. It is an original map that we made, has not been published elsewhere, and contains only basic shapefiles of the outlines of Canada and Alberta from ArcMap, and points delineating the locations of ABMI camera trap sites.*

*Figure 2 is modified from the reference cited, which contains the text “Use of this Material: This publication may be reproduced in whole or in part and in any form for educational, data collection or non-profit purposes without special permission from the ABMI, provided acknowledgement of the source is made”.*

*We therefore do not feel any further permission is needed for the figures included in our manuscript.*

Reviewers' comments:

**Comments to the Author**

Reviewer #1: This is an interesting paper that measures the effect of lures on the detection rate of multiple species and functional groups across a wide region of the province of Alberta in Canada. The paper is well written and the questions and methods are clearly described.

*Thank you for your positive feedback and helpful comments.*

I only have a few suggestions/comments:

- Can the authors provide a hyperlink pointing to the data set? I did not see in the main manuscript.

*We updated the data accessibility information (above and in revised cover letter) to include a direct link to the ABMI camera trap data (*[*https://abmi.ca/home/data-analytics/da-top/da-product-overview/remote-camera-mammal-data/remote-camera-mammal-data-download.html*](https://abmi.ca/home/data-analytics/da-top/da-product-overview/remote-camera-mammal-data/remote-camera-mammal-data-download.html)*)*

- Please justify the GLMM approach with a zero inflated binomial link.

*We included a better description and justification of the modelling approach on lines 158-163. Specifically, we explained why a negative binomial distribution was used (non-normal, overdispersed count data that were better fit by NB than by Poisson) and that there was a high proportion of zero counts (justifying the zero inflation).*

- The camera traps within a site seem relatively close to each other for many of the species studied. Was spatial autocorrelation in detection rate modeled for each species?

*We included a random effect for site to account for potential non-independence among camera traps within a site. We did not model spatial autocorrelation between sites as they were separated by at least 20 km. Even within sites, the distance between camera traps (min. 600 m) has been shown by other camera trap studies to result in little or no spatial autocorrelation (e.g. Kays et al. 2011. Camera traps as sensor networks for monitoring animal communities. International Journal of Research and Reviews in Wireless Sensor Networks 1:19-29.)*

- Please include a table with values of all parameters for each model, including standard errors. Some of the parameters are mentioned in the MS, but the information needs to be included in a table for easy inspection.

*We added a supplementary table (S5_Table) which includes the parameter estimates. It is cited in the Results on lines 201 and 215.*

- What do the authors recommend people do when comparing between camera trap studies with different levels of lure or scent, specially when modeling population trends over time? Does it make sense to include lure as a covariate at the camera trap sample level per species? or functional group?

*This is a great question reflecting a key challenge in comparing CT studies using different protocols. We have now incorporated this particular recommendation on lines 283-5 of the revised manuscript, as well as the recommendation to consider occupancy modelling (next comment).*

- Another possible approach is to model occupancy and detection probability for each species with lure as a covariate (and site as a random effect). What would the authors predict in this case? My suspicion is that lure affects the detection probability for certain species but not the underlying population metrics. Please discuss and put into a wider context.

*We added the recommendation to consider occupancy modelling to our general call for further analytical work to address the variable effects of lure (lines 284-288, including a citation to the key reference book on occupancy modelling by MacKenzie et al. 2018).*

*We did not test occupancy models in this paper, partly due to our concerns with their interpretation across multispecies surveys (e.g. sensitivity to differences in species movements and the violation of the assumption of site closer, see Neilson et al 2018. Animal movement affects interpretation of occupancy models from camera-trap surveys of unmarked animals. Ecosphere 9:e02092). But we do agree it would be an interesting analysis to investigate in future research.*

Hope these comments are useful and congratulations for a great study!

*Thank you!*
